# The novel human astrovirus VA1 requires the proteasome during cell entry

**DOI:** 10.1099/jgv.0.002163

**Published:** 2025-10-07

**Authors:** Luis E. Jiménez, Susana López, Carlos F. Arias, Tomás López

**Affiliations:** 1 Departamento de Genética del Desarrollo y Fisiología Molecular, Instituto de Biotecnología, Universidad Nacional Autónoma de México, Cuernavaca, Mexico

**Keywords:** cell entry, human astrovirus, proteasome, VA1

## Abstract

Astroviruses are important aetiological agents of gastroenteritis. Recently, two novel human astrovirus (HAstV) clades, VA and MLB, have been identified. However, the replication cycle of these viruses remains poorly characterized. Among these, the novel astrovirus VA1 has been of particular interest due to its reported association with neurological disease in immunocompromised patients. Previous studies have demonstrated that a functional proteasome is essential for the efficient replication of classic HAstVs. In this study, we investigated the role of the proteasome in the replication of HAstV-VA1. We assessed the impact of two proteasome inhibitors, MG132 and bortezomib, on viral replication. Both inhibitors significantly reduced viral protein and infectious progeny production in a dose-dependent manner. Our findings indicate that the inhibitory effects of these compounds are mediated through a mechanism affecting virus entry and a post-entry step in the viral replication cycle during the virus replication cycle.

## Introduction

Astroviruses are single-stranded, positive-sense RNA viruses belonging to the family *Astroviridae*. This family is divided into two genera: *Avastrovirus*, which infects avian species, and *Mamastrovirus*, which infects mammals, including humans. Human astroviruses (HAstVs) are well-established aetiologic agents of gastroenteritis in young children [1]. They exhibit considerable genetic diversity and are classified into three clades. The first described, referred to as classic astroviruses, comprises eight serotypes, with human serotype 1 (HAstV-1) being the most prevalent [1]. The remaining two clades, referred to as non-classic astroviruses, are genetically more closely related to animals than to HAstVs [2]. They were identified ~15 years ago using high-throughput sequencing techniques. While initially considered gastrointestinal pathogens, studies have since questioned their direct association with gastroenteritis [3].

Recent reports have implicated these genetically divergent astroviruses in cases of meningitis and encephalitis, particularly in immunocompromised patients. Of the 17 documented cases of neurological disease linked to astrovirus infection, 10 were attributed to HAstV-VA1 [4,5]. HAstV-VA1 was originally isolated from the faeces of children, with only one isolate successfully adapted to cell culture [6,7]. Notably, HAstV-VA1 differs from classical HAstV in several aspects of its replication cycle, including its independence from trypsin activation and its intracellular capsid processing in a caspase-independent manner [7,8].

The proteasome, a critical component of the ubiquitin-proteasome system (UPS), is the main protein degradation machinery in cells. The UPS regulates protein stability and is involved in various cellular processes [9]. Viral manipulation of the UPS has been reported to have both pro- and antiviral effects [9,10]. In the context of astroviruses, we previously demonstrated that proteasome inhibition reduces the production of viral progeny by impairing viral RNA and protein synthesis of HAstV-8 [11].

In this study, we characterized the role of proteasome activity in HAstV-VA1 replication, focusing on the impact of two chemical proteasome inhibitors. Our findings indicate that the activity of the proteasome is necessary for efficient virus entry and is also required at a post-entry step of virus replication.

## Methods

### Cells, Viruses and Reagents

The C2BBe1 cell line, a clone derived from the cell line Caco2, was obtained from ATCC. The HAstV-VA1 strain was kindly provided by Dr D. Wang (Washington University, St Louis, Missouri). Virus stocks were propagated in C2BBe1 cells and stabilized with 3% sucrose. Proteasome inhibitors MG132 and bortezomib were purchased from Santa Cruz Biotechnology (Cat. No. sc-201220 and sc-217785). Stock solutions were prepared in DMSO at a concentration of 10 mM. Monoclonal anti-tubulin antibody was obtained from Santa Cruz Biotechnology (Cat. No. sc-51500).

### LDH release assay

For the lactate dehydrogenase (LDH) release assay, we used the In Vitro Toxicology Assay, Lactic Dehydrogenase-based kit from Sigma (Cat No. TOX-7). Cells were incubated with the indicated concentration of drugs for 24 h. At this time, the medium was collected, the cells attached to the plate were lysed and LDH activity was determined following the manufacturer’s instructions.

### Polyclonal serum production

A recombinant protein corresponding to amino acids 441–595 of the HAstV-VA1 NSP1a sequence (GenBank Accession: NC_013060) was expressed in *Escherichia coli*. The viral protease sequence was cloned into the pGEX-4T vector using the EcoRI and XhoI restriction sites. The following primers were employed for RT-PCR amplification of the viral RNA: Forward: 5′-GCATGGAATTCAACAGCTA-3′ and reverse: 5′-GCATACTCGAGGGTGGTAA-3′. To obtain anti-protease hyperimmune sera, New Zealand rabbits were immunized with 250 µg of recombinant protein. The initial dose was administered with Freund’s complete adjuvant, followed by three booster immunizations at 2 week intervals using the same amount of protein in Freund’s incomplete adjuvant. The production of anti-HAstV-VA1 polyclonal sera has been previously described [12].

### Infectivity assay

The infectivity assay was performed as previously described, with modifications [8]. Briefly, C2BBe1 cells were grown to confluence in 96-well plates and infected with 50 µl of twofold serial dilutions of viral lysates for 1 h at 37 °C. Following infection, the unbound virus was removed, and the cells were incubated for 24 h for HAstV-VA1 or 18 h for HAstV-8 at 37 °C. Cell monolayers were then fixed with 2% formaldehyde for 20 min at room temperature, washed twice with PBS and permeabilized with 0.2% Triton X-100 for 15 min. Viral foci were detected using an immunoperoxidase assay as previously described [11,12]. The number of f.f.u. was determined using a Nikon TMS inverted phase-contrast microscope equipped with a 20× objective.

### Western blot analysis

C2BBe1 cells were seeded in 24-well plates and grown to confluence before infection with HAstV-VA1 at an m.o.i. of 10. At the indicated times post-infection, cells were treated with either MG132, bortezomib or DMSO (vehicle control). At 24 h post-infection (hpi), cells were lysed with Laemmli sample buffer and subjected to SDS-PAGE on 12.5% polyacrylamide gels. Proteins were transferred to NC membranes as described previously [9], and membranes were incubated with the indicated antibodies, using Alexa 647-conjugated anti-rabbit or anti-mouse secondary antibodies. Protein signals were visualized using a Typhoon FLA 9500 scanner (GE Healthcare).

### RNA transfection

For RNA extraction, C2BBe1 cells were grown to confluence in 6-well plates and infected with HAstV-VA1 or HAstV-8 at an m.o.i. of 10. At 24 hpi, total RNA was extracted using TRIzol reagent (Invitrogen, Cat. No. 1559026) according to the manufacturer’s instructions. For RNA transfection, C2BBe1 cells were seeded in 96-well plates and grown to confluence. Cells were then incubated with a transfection mixture containing 140 ng of total RNA and 3 µl of Lipofectamine 3000 (Invitrogen, Cat. No. 31985062) in a final volume of 50 µl of Opti-MEM for 1 h at 37 °C. In parallel, cells were infected with HAstV-VA1 or HAstV-8 at an m.o.i. of 0.02. Proteasome inhibitors were added at the indicated concentrations for 6 h, and cells were extensively washed to remove residual drug. At 24 h post-transfection (hpt) for HastV-VA1 or 18 hpt for HAstV-8, cells were fixed and processed for infectivity as described.

### Statistical analysis

Statistical analysis and non-linear regression were performed using GraphPad Prism 9.0. Statistical significance was calculated using a t-test.

## Results

**Fig. 1.**
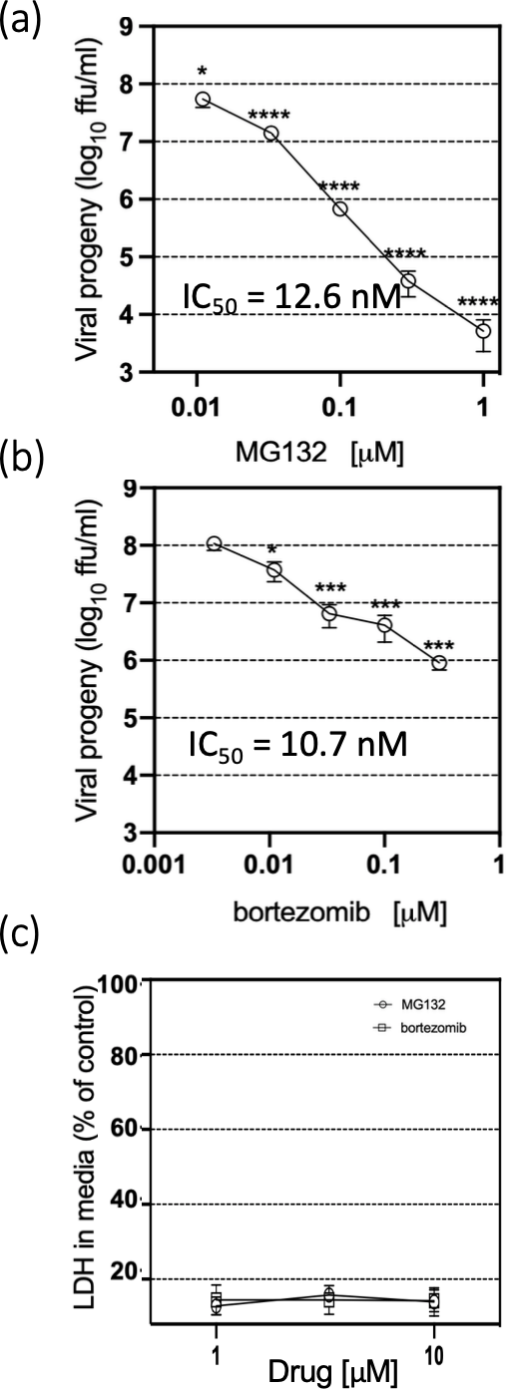
Proteasome inhibitors reduce viral progeny production. C2BBe1 cells were infected with HAstV-VA1 at an m.o.i. of 10. Following the adsorption period, cells were treated with the indicated concentrations of (a) MG132 or (b) bortezomib. At 24 hpi, cells were collected, and viral progeny production was assessed using an immunoperoxidase assay [9]. Data are presented as the log₁₀ of f.f.u. ml^−1^ and represent the mean±sd of three independent experiments performed in duplicate. Statistical significance was evaluated by a t-test *, ≤0.05; ***, ≤0.001; ****, ≤0.0001. IC_50_ value was calculated using non-linear regression. (**c**) Uninfected C2BBe1 cells were treated with the indicated concentrations of MG132 or bortezomib for 24 h. Media and cell lysates were collected separately, and LDH release was measured as an indicator of cytotoxicity. The results are expressed as the percentage of LDH activity in the media relative to total LDH and represent the mean±sd of three independent experiments performed in duplicate.

To assess whether proteasome activity is involved in HAstV-VA1 progeny production, C2BBe1 cells were infected at an m.o.i. of 10 for 1 h at 37 °C. After the adsorption period, the unbound virus was removed, and the cells were treated with different concentrations of the proteasome inhibitors MG132 or bortezomib, which were kept for 24 h. The cells were harvested, and cell lysates were analysed for viral progeny production (Fig. 1a, b). MG132 treatment resulted in a dose-dependent reduction in the infectious viral progeny, with a four-log decrease observed at 1 µM and a one-log decrease at 33 nM (Fig. 1a). Similarly, bortezomib treatment led to a two-log reduction at 300 nM and a one-log reduction at 100 nM (Fig. 1b). Using non-linear regression, we calculated that the IC_50_ for MG132 and bortezomib was 12.6 and 10.7 nM, respectively. To assess potential cytotoxic effects, LDH release was measured at 24 hpi. Neither MG132 nor bortezomib induced cytotoxicity in C2BBe1 cells at the tested concentrations (Fig. 1c).

**Fig. 2.**
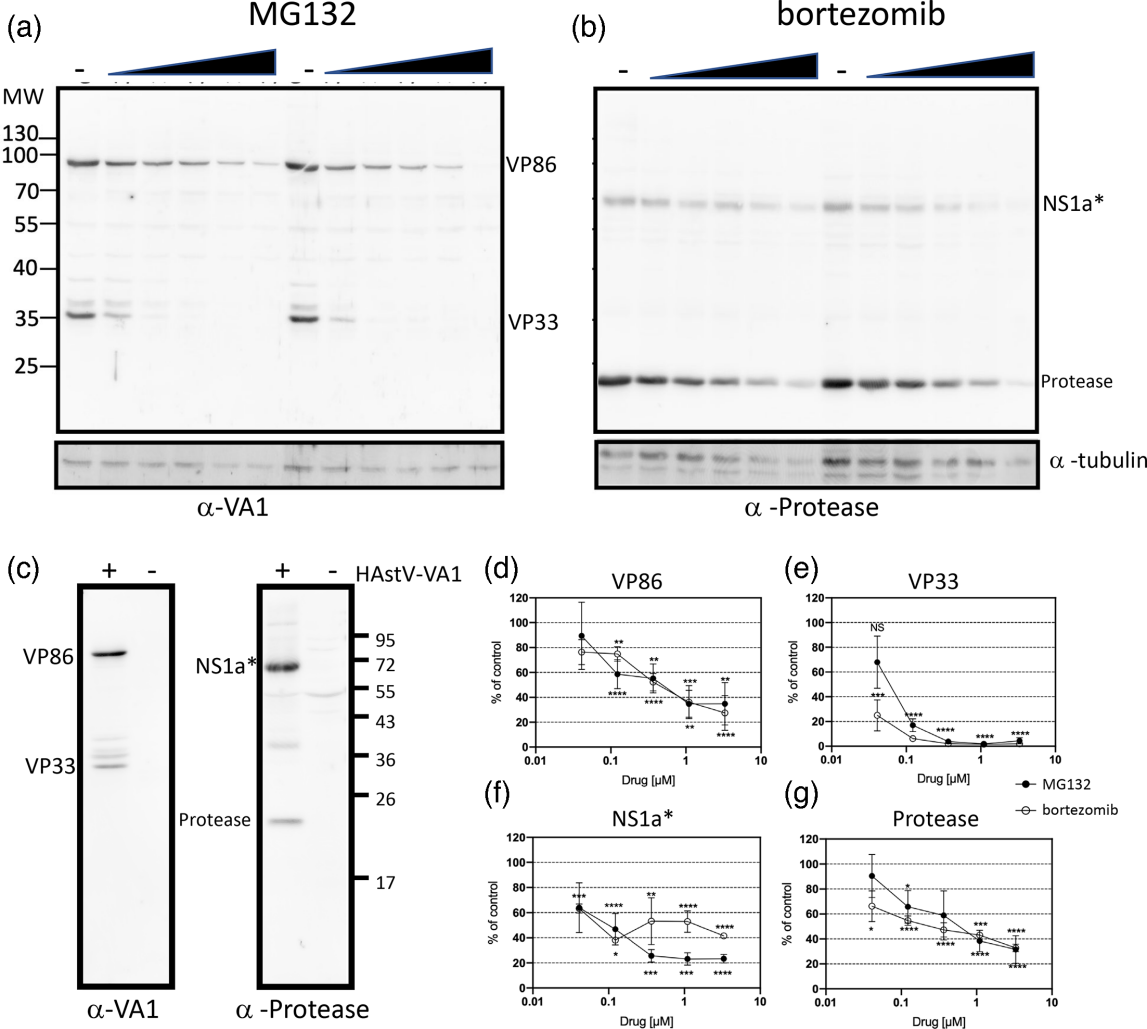
Proteasome inhibitors reduce the accumulation of viral proteins. C2BBe1 cells were infected with HAstV-VA1 at an m.o.i. of 10. Following the adsorption period, cells were treated with the indicated concentrations of MG132 or bortezomib. At 24 hpi, cells were harvested, and viral protein expression was analysed by Western blotting. (**a**) Detection of capsid protein using anti-HAstV-VA1 hyperimmune serum (upper panel) or tubulin (bottom panel). (**b**) Detection of viral protease using anti-HAstV-VA1 protease polyclonal serum (upper panel) or anti-tubulin (bottom panel). (**c**) Control western blot compared infected and mock-infected cells with the indicated antibodies. (**d**) Densitometric analysis of expression of capsid protein precursor (VP86). (**e**) Densitometric analysis of expression of viral core (VP33). (**f**) Densitometric analysis of expression of non-structural protein precursor. (**g**) Densitometric analysis of expression of viral protease. Data are analysed from three independent experiments and represent the mean±sem. Statistical significance was evaluated by a t-test *, ≤0.05; **, ≤0.01; ***, ≤0.001; ****, ≤0.0001.

To characterize the level of viral proteins produced in the presence of the inhibitors, cells were infected with HAstV-VA1 at an m.o.i. of 10, treated with the indicated concentration of the drugs, and at 24 hpi, the accumulation of viral proteins was evaluated by Western blot. Both proteasome inhibitors reduced the accumulation of the virus capsid proteins as detected with a polyclonal serum directed to the purified virus (Fig. 2a, d and e). To further analyse their impact on the non-structural proteins, a polyclonal serum against the viral protease was generated (see Methods). This antibody detected two major products: a band of ~70 kDa, corresponding to one polyprotein protease precursor. This precursor is most likely the result of co-translational processing of nsp1a/1 and represents a polyprotein that includes ns1a/2-ns1a/3-ns1a/4 (NS1a*), as well as a second band of ~24 kDa, representing the mature protease (Fig. 2b, c). Additionally, with anti-protease antibody, a non-specific 55 kDa band was observed in both infected and non-infected cells, and other minor bands, which could represent different levels of precursor processing, were observed (Fig. 2c). Both MG132 and bortezomib significantly reduced the expression of the mature protease and of the polyprotein protease precursor (Fig. 2b, f and g). As previously demonstrated, under the conditions used in these experiments, the host cell protein synthesis is not affected, suggesting that the effect of proteasome inhibitors is specific for viral proteins [11]. These findings suggest that active proteasome function is required for efficient HAstV-VA1 replication in C2BBe1 cells, affecting both viral protein accumulation and the production of viral progeny.

**Fig. 3.**
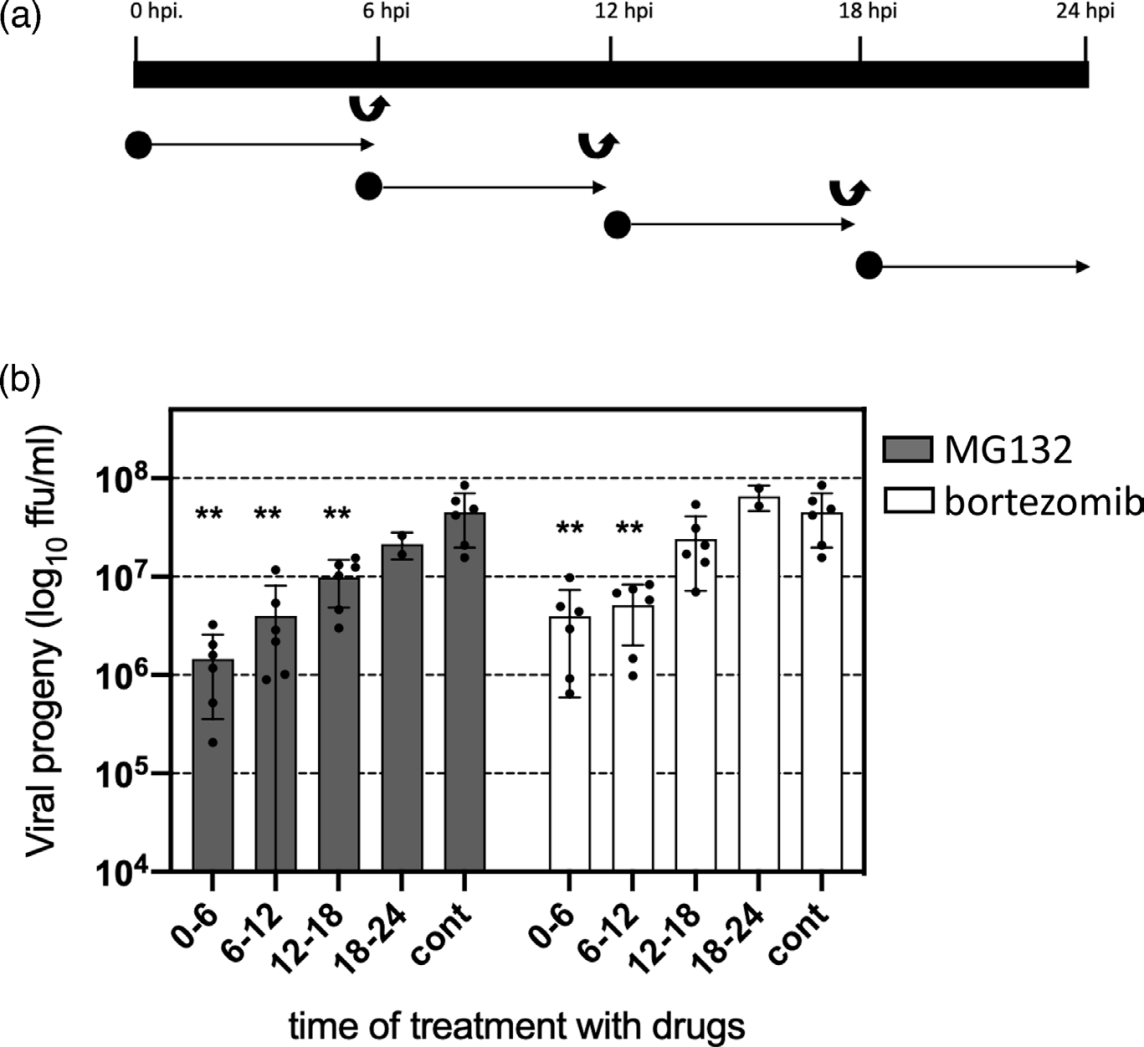
Proteasome inhibitors affect an early step in the HAstV-VA1 replication cycle. (**a**) Schematic representation of the experimental design. Cells were infected with HAstV-VA1 at an m.o.i. of 10. At the indicated times post-adsorption, either MG132 (300 nM) or bortezomib (100 nM) was added for 6 h and subsequently removed by washing. Black circles indicate the time points at which the inhibitors were applied, and curved arrows indicate the time of drug removal. At 24 hpi, cells were harvested, and viral progeny production was quantified using a peroxidase-based focus-forming assay [12]. (**b**) C2BBe1 cells were infected with HAstV-VA1 at an m.o.i. of 10 and treated as described in panel (**a**). At 24 hpi, cells and media were collected, and viral progeny production was assessed by a peroxidase foci-forming assay [12]. Data are presented as the log₁₀ of f.f.u. ml^−1^ and represent the mean±sd of three independent experiments conducted in duplicate. Statistical significance was evaluated by a t-test **, ≤0.01.

To determine whether the proteasome-dependent event occurs early in the replication cycle, we infected cells with HAstV-VA1 at an m.o.i. of 10. At different times post-infection, proteasome inhibitors were added during 6 h periods, as indicated (Fig. 3a). The inhibitory effect of both drugs diminished when their addition was delayed after viral adsorption, suggesting that the proteasome-dependent event occurs early in the HAstV-VA1 replication cycle (Fig. 3b). Considering these results, we next investigated whether viral entry was affected.

**Fig. 4.**
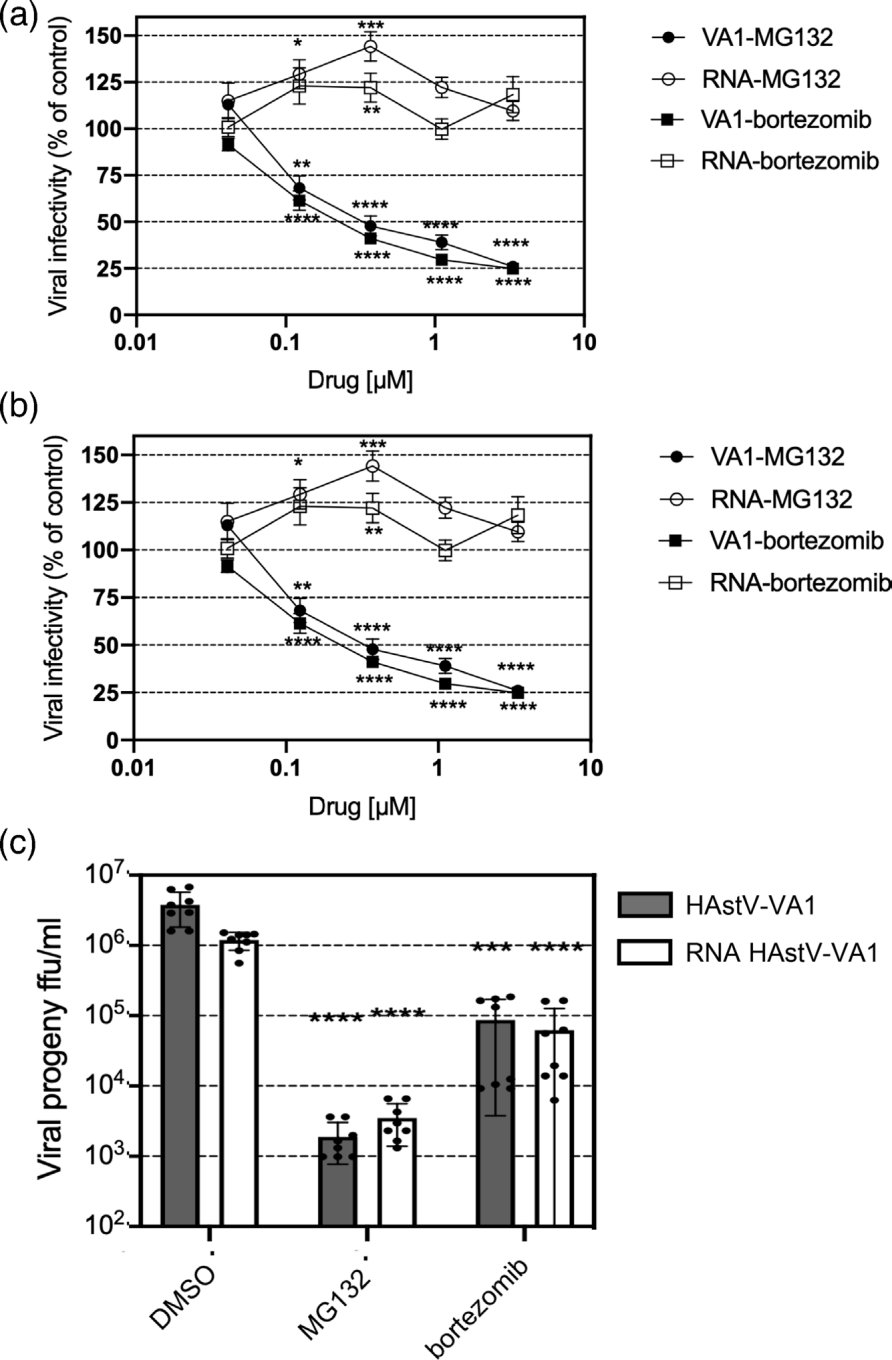
Proteasome inhibitors reduce HAstV-VA1 infectivity but do not affect viral foci formation from transfected viral genomic RNA. Confluent monolayers of C2BBe1 cells were either infected with (a) HAstV-VA1 or (b) HAstV-8 at an m.o.i. of 0.02 or transfected with viral RNA in the presence of the indicated concentrations of MG132 or bortezomib for 6 h. At 24 hpi, cells for HAstV-VA1 or 18 hpi for HAstV-8 were fixed and stained for viral foci detection using an immunoperoxidase assay [11,12]. Data are expressed as f.f.u. ml^−1^ and represent the mean±sd of four independent experiments conducted in duplicate. (**c**) Cells were infected with HAstV-VA1 at an m.o.i. of 0.02 or RNA transfected for 1 h; the latter cells were incubated with MG132 at 300 nM or bortezomib at 100 nM. At 24 hpi, cells were collected and processed for viral progeny production. Data are presented as the log₁₀ of f.f.u. ml^−1^ and represent the mean±sd of three independent experiments performed in duplicate. Statistical significance was evaluated by a t-test ****, ≤0.0001. IC_50_ values were calculated using non-linear regression.

To determine whether viral entry in HAstV-VA1 is proteasome-dependent, we compared the effects of MG132 and bortezomib on viral foci formation following either virus infection or transfection with viral genomic RNA. The rationale for this approach was that if the inhibitors affected virus entry, bypassing this step through genomic RNA transfection would prevent any reduction in the viral foci formation. For this, cells were infected at an m.o.i. of 0.02 or transfected with total RNA as indicated in Methods. As shown in Fig. 4(a), both MG132 and bortezomib significantly reduced the number of viral infectious foci detected in a dose-dependent manner. However, when viral RNA was directly transfected into cells, neither inhibitor had an impact on the infectivity of the virus, suggesting that the proteasome activity is required for the entry of HAstV-VA1 into host cells. Previous studies with HAstV-8 indicated that proteasome activity was required for production of positive-sense viral RNAs [11], but direct evaluation of its effect on virus entry was not evaluated. As shown in Fig. 4(b), similar to HAstV-VA1, the entry of HAstV-8 is inhibited by proteasome inhibitors. For viral entry, the IC_50_ for both inhibitors is in the nanomolar range (Fig. 4a, b).

To determine if, in addition to viral entry, the proteasome inhibitors affect a post-entry viral replication event, we compared the effect of MG132 (300 nM) and bortezomib (100 nM) on viral progeny production from infected or RNA-transfected cells. As shown in Fig. 4(c), both inhibitors reduced the production of viral progeny of HAstV-VA1-infected or RNA-transfected cells to a similar extent, indicating that the proteasome is necessary for producing viral progeny in a post-entry step of the virus replication cycle.

## Discussion

In this study, we investigated the effect of two proteasome inhibitors, MG132 and bortezomib, on HAstV-VA1 replication. Both inhibitors significantly reduced viral progeny production, with MG132 exhibiting a more pronounced effect than bortezomib. This differential impact may be attributable to the broader inhibitory profile of MG132, which targets not only the proteasome but also other cellular proteases such as *m*-calpain [13] and cathepsin-l [14], as well as certain viral proteases [14,15]. In contrast, bortezomib is a more selective proteasome inhibitor and was the first FDA-approved proteasome inhibitor for the treatment of multiple myeloma [16,17]. Considering the specificity of bortezomib and the fact that it caused a two-log reduction in the production of viral progeny at 300 nM (Fig. 1b), this result strongly suggests that the activity of the proteasome is critical for HAstV-VA1 replication. This finding aligns with previous studies that identified the proteasome as a potential antiviral target, including the replication of SARS-CoV-2 [18].

The UPS has been implicated in various stages of the life cycle of several viruses, including virus entry [19]. For instance, dengue [20], influenza [21] and herpesvirus [22] require an active UPS for successful entry into the cell. In the present study, proteasome inhibition effectively suppressed HAstV-VA1 infectivity when the inhibitors were applied within the first 6 hpi. The 6 h incubation period was chosen to encompass the uncoating phase, since the time of this phase for HAstV-VA1 is currently unknown. For classic HAstV-8, the half-time for RNA release is ~130 min [23]. Given our previous findings that HAstV-VA1 has a longer eclipse period than HAstV-Yuc8 during its replication cycle [8], a 6 h window was considered adequate to capture the key entry events, including capsid disassembly and genome release. This conclusion is supported by the fact that transfection of the HAstV-VA1 genomic RNA into cells, bypassing the virus’s cell entry process, abolishes the inhibitory effect of both drugs. Of interest, our data confirm that the effect of proteasome inhibitors on virus entry is common for classical (HAstV-8) and VA1 astroviruses (Fig. 4a, b).

Our findings indicate that proteasome proteolytic activity is required during the early stages of HAstV-VA1 replication, specifically during virus entry. However, a post-entry effect was confirmed by the fact that proteasome inhibitors reduce viral progeny in RNA-transfected cells (Fig. 4c). Further studies are needed to determine whether proteasome activity is required for virus binding, internalization or genome release into the cytoplasm and to identify the specific step(s) post-entry affected by proteasome inhibitors. The differences observed in the effect between the non-structural proteins and the mature core (VP33) suggest a role of the proteasome in capsid processing (Fig. 2a, b). The antiviral potency of MG132 and bortezomib on viral progeny and infectivity appears comparable (Figs1  4). Nevertheless, MG132 exerts a more pronounced inhibitory effect on progeny production (Fig. 1), suggesting that its broader action on additional proteases may interfere with post-entry steps of viral replication [13,15]. Elucidating the specific step(s) at which the proteasome exerts its effect on HAstV infection will enhance our knowledge about the biology of this important pathogen.

## Abbreviations

HAstVs: human astroviruses
hpi: h post-infection
hpt: h post-transfection
LDH: lactate dehydrogenase
UPS: ubiquitin-proteasome system
